# Fungal Endophthalmitis on Ocular Ultrasound: A Case Report

**DOI:** 10.5811/cpcem.2021.10.53797

**Published:** 2022-01-28

**Authors:** Kimberly Fender, Merrick Bautista, Hiten Patel, Benjamin Ostro, Creagh Boulger

**Affiliations:** The Ohio State University Wexner Medical Center, Department of Emergency Medicine, Columbus, Ohio

**Keywords:** point-of-care ultrasound, POCUS, ocular, endophthalmitis, case report

## Abstract

**Introduction:**

Endophthalmitis is a rare intraocular infection caused by numerous organisms from several possible sources. Fungal endophthalmitis is a rare subset of this pathology with limited diagnostics available. One of the few options to make this diagnosis is vitreous sampling, which is invasive, and results are not immediately available.

**Case Report:**

This case report describes the successful use of point-of-care ultrasound to visualize an intraocular fungal mass in a 60-year-old male who presented to the emergency department (ED) with two weeks of left eye pain and erythema approximately two months postoperative from a cataract extraction surgery.

**Conclusion:**

Fungal endophthalmitis is a rare and challenging diagnosis. Methods of diagnosing this pathology are not readily available in the ED. Point-of-care ultrasound may be a useful adjunct for the prompt diagnosis of fungal endophthalmitis.

## INTRODUCTION

Endophthalmitis refers to any bacterial or fungal infection of the vitreous or aqueous humor. Endophthalmitis is a rare but serious intraocular infection that can result in irreversible vision loss in a matter of hours.^1^ The infection can originate from an external source, such as surgery or trauma, or from an endogenous source by way of hematologic spread.^3^ Fungal endophthalmitis occurs rarely and is difficult to diagnose, with often non-specific presentations, and has a poor prognosis.^4^ Prompt diagnosis and treatment are of the utmost importance. While point-of-care-ultrasound (POCUS) is frequently used in the emergency department (ED) to diagnose emergent ophthalmologic conditions such as vitreous hemorrhage, retinal detachment, lens dislocation, and retained intraocular foreign body, it is less commonly used to diagnose endophthalmitis, and there is limited literature regarding diagnostic capabilities in the ED for this condition.^1^,^2^ This case report describes the successful use of POCUS to visualize fungal endophthalmitis in a patient with a history of cataract extraction surgery who presented to the ED with a complaint of left eye blurry vision.

## CASE REPORT

A 60-year-old male with a past medical history of cataracts, hypertension, and insulin-dependent diabetes mellitus presented to the ED with a chief complaint of left eye blurred vision. He was 10 weeks postoperative from a left eye cataract extraction surgery. Three weeks prior to his ED presentation, he had noticed a vague left eye ache associated with redness and worsening blurry vision. He denied fever, photophobia, and eye discharge.

The patient initially presented to an ophthalmologist for evaluation of his symptoms. Five days prior to his ED visit a fluid sample was collected from the anterior chamber of his left eye. Retinal imaging revealed mild macular edema and mild vitritis. Intravitreal ceftazidime and vancomycin were administered with an initial concern for endophthalmitis, and he was prescribed ofloxacin drops. He returned for follow-up the next day endorsing mild improvement of his left eye pain, and he was additionally started on prednisone drops. Three days later he returned for another follow-up appointment with progressive vision loss, and he received intravitreal amphotericin B. Despite this treatment, he re-presented to the clinic the following day with progressively worsening vision. Ophthalmologic exam revealed worsening inflammation and new hypopyon. With this progression of symptoms, he was instructed to present to the ED for further ophthalmologic evaluation and admission.

Vital signs on presentation to the ED were a temperature of 97.1° Fahrenheit, blood pressure 170/88 millimeters mercury, heart rate of 70 beats per minute, a respiratory rate of 20 breaths per minute, and an oxygen saturation of 99% on room air. Physical exam was notable for left eye conjunctival injection and chemosis, Pupils were equally round and reactive bilaterally, extraocular movements were intact and performed without additional discomfort, and intraocular pressures were within normal limits. Visual acuity was 20/25 on the right and 20/70 on the left.

Point-of-care ultrasound of his left eye revealed a mobile, hyperechoic spherical mass with an anechoic center in the posterior chamber. The mass was approximately 0.5 centimeters in diameter. Additional intraocular ultrasound views revealed multiple hypoechoic structures stemming from the lateral aspects of the mass. No clear anchoring was seen to the retina, no retinal detachment was seen, nor was there any dilation of the optic nerve.

The patient was admitted to the hospital for continued workup and management where blood cultures, aqueous cultures, and cultures of tubular angle mass aspirate were negative. The patient was ultimately discharged with a presumptive diagnosis of fungal endophthalmitis based on his acute clinical deterioration after initiation of steroids and lack of bacterial culture growth. Two weeks after discharge he was improving but still had some blurry vision and was taken back to the operating room for another vitreous washout and intraocular antifungals. His cultures from this operation were also negative, bacterial, viral, and fungal. Two weeks after this washout his visual acuity returned to 20/25 in his left eye, and he reported resolution of the pain, blurry vision, and floaters in his visual field.

## DISCUSSION

Endophthalmitis is a serious intraocular infection that can lead to irreversible vision loss within a few hours. Classically, this infection occurs following ocular surgery, eye trauma, or systemic bacteremia or fungemia.^1^ Patients may present with any combination of unilateral eye pain, floaters, blurry vision, and photophobia; however, it may be asymptomatic, creating a diagnostic challenge for providers. Thus, the prompt diagnosis and treatment of this uncommon condition is of the utmost importance to preserve visual function and eye structure. Unfortunately, despite the seriousness of this condition, there are few tools available to aid providers in the diagnosis of this infection.^1^

**CPC-EM Capsule**

What do we already know about this clinical entity?

*Fungal endophthalmitis is an uncommon, serious ocular infection with a non-specific presentation; it is difficult to diagnose and treat*.

What makes this presentation of disease reportable?

*This case demonstrates the possible utility of ultrasound in making this challenging diagnosis*.

What is the major learning point?

*Ocular ultrasound can be useful in the diagnosis of fungal endophthalmitis. The lesion may appear as a hyperechoic mass in the posterior chamber*.

How might this improve emergency medicine practice?

*Using point-of-care ultrasound could expedite time to diagnosis and treatment of a disease with significant complications that is challenging to diagnose*.

The diagnosis of endophthalmitis requires a thorough ophthalmologic evaluation, intraocular and blood cultures, and imaging such as computed tomography or radiographs to evaluate for endogenous sources.^1^ The variation of presentations and lack of a diagnostic protocol pose a challenge for providers, especially in the ED. One study reviewing endophthalmitis found that up to 30% of cases are asymptomatic at the time of diagnosis.^5^ Another study revealed that intraocular cultures were positive in only 41.7% of cases and in 28.6% of cases in which treatment already had been started.^6^ Blood cultures provided little diagnostic benefit and were positive in only 48.5% of these cases.^6^ There are no clear treatment guidelines regarding the use of intravenous or intravitreal antimicrobials or vitrectomy.^7^ Regardless of the vast differences in diagnostics and treatments, the visual outcome of endophthalmitis tends to be poor.^1^

**Image 1:**
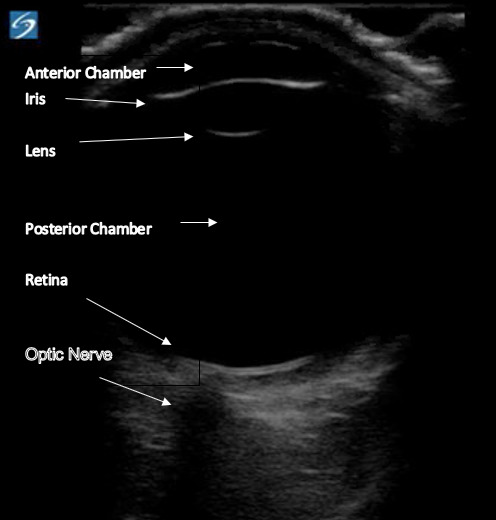
Normal ocular ultrasound with relevant anatomy.

While ocular ultrasound is commonly used in the ED to evaluate retinal detachment, vitreous hemorrhage, and papilledema, there is limited literature regarding its utility in evaluating patients for suspected endophthalmitis. One case report using POCUS focused on evaluating the presence of hyperechoic vitreous debris while another article focused on hyperechoic, membranous material in the vitreous humor.^8^,^9^ Image 1 demonstrates a normal ocular ultrasound with the important anatomy labeled.

**Image 2:**
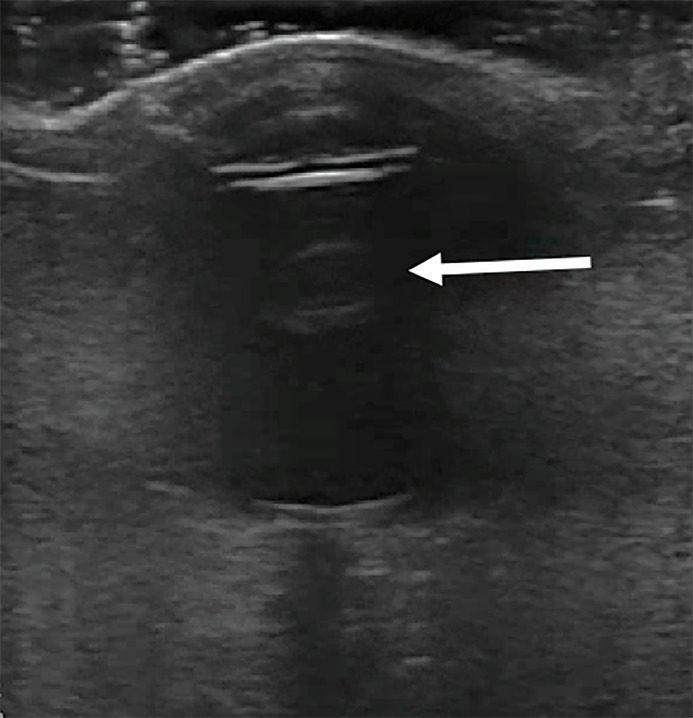
Hyperechoic spherical structure seen in the posterior chamber of the left eye noted at the tip of the arrow.

**Image 3:**
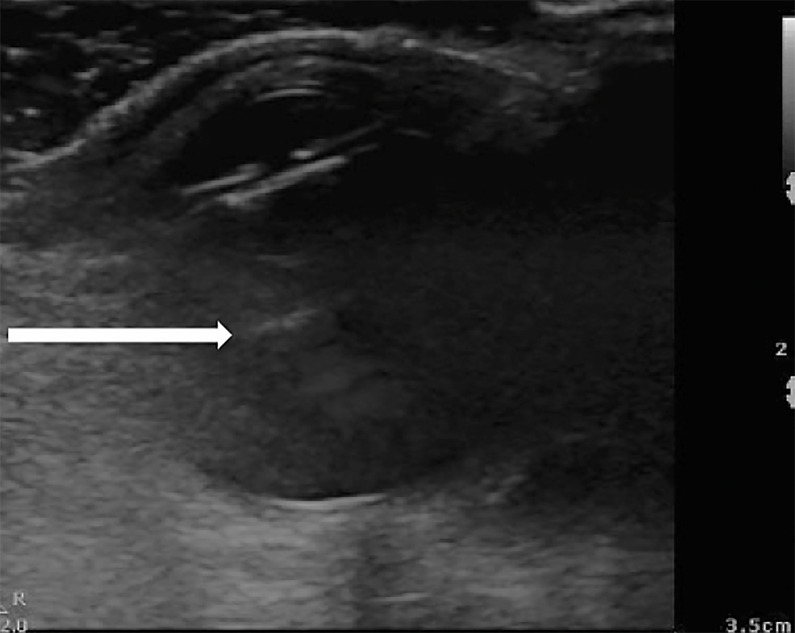
Hypoechoic anchoring of previous spherical structure as indicated at the tip of the arrow.

Upon literature review, we found no identifiable case reports describing the presence of a hyperechoic circular mass in the vitreous humor related to the diagnosis of endophthalmitis (Image 2). Additionally, some hypoechoic material is visible in Image 3 and the supplemental video, which could be anchoring the structure laterally; however, this is not clearly visualized on all images. While these images alone are insufficient to diagnose endophthalmitis, the visualized mass on POCUS is strongly suspected to be related to the patient’s left eye symptoms. Given the minimal number of tools for diagnosing endophthalmitis, POCUS findings of a hyperechoic mass within the vitreous should be considered as a useful adjunct for timely diagnosis.

## CONCLUSION

Fungal endophthalmitis is an uncommon, serious ocular infection with a non-specific presentation, making it difficult to diagnose and treat. This case report demonstrates the use of POCUS in the ED as an adjunct in the diagnosis of this condition. While ultrasound findings are not definitive for the diagnosis of endophthalmitis, there may be some benefit in using POCUS findings in the ED to aid in the prompt diagnosis and treatment of this devastating condition, which could potentially lead to a better visual outcome.

## Supplementary Information

Video Point-of-care ultrasound demonstrating a hyperechoic spherical structure in the posterior chamber of the left eye noted in the center of the red circle.
